# A Perspective on a Quality Management System for AI/ML-Based Clinical Decision Support in Hospital Care

**DOI:** 10.3389/fdgth.2022.942588

**Published:** 2022-07-06

**Authors:** Richard Bartels, Jeroen Dudink, Saskia Haitjema, Daniel Oberski, Annemarie van ‘t Veen

**Affiliations:** ^1^Digital Health, University Medical Center Utrecht, Utrecht University, Utrecht, Netherlands; ^2^Department of Neonatology, Wilhelmina Children's Hospital, University Medical Center Utrecht, Utrecht University, Utrecht, Netherlands; ^3^Brain Center Rudolf Magnus, University Medical Center Utrecht, Utrecht University, Utrecht, Netherlands; ^4^Central Diagnostic Laboratory, University Medical Center Utrecht, Utrecht University, Utrecht, Netherlands; ^5^Department of Medical Microbiology, University Medical Center Utrecht, Utrecht University, Utrecht, Netherlands

**Keywords:** AI, machine learning (ML), clinical decision support, implementation, quality management system, ISO15189

## Abstract

Although many artificial intelligence (AI) and machine learning (ML) based algorithms are being developed by researchers, only a small fraction has been implemented in clinical-decision support (CDS) systems for clinical care. Healthcare organizations experience significant barriers implementing AI/ML models for diagnostic, prognostic, and monitoring purposes. In this perspective, we delve into the numerous and diverse quality control measures and responsibilities that emerge when moving from AI/ML-model development in a research environment to deployment in clinical care. The Sleep-Well Baby project, a ML-based monitoring system, currently being tested at the neonatal intensive care unit of the University Medical Center Utrecht, serves as a use-case illustrating our personal learning journey in this field. We argue that, in addition to quality assurance measures taken by the manufacturer, user responsibilities should be embedded in a quality management system (QMS) that is focused on life-cycle management of AI/ML-CDS models in a medical routine care environment. Furthermore, we highlight the strong similarities between AI/ML-CDS models and *in vitro* diagnostic devices and propose to use ISO15189, the quality guideline for medical laboratories, as inspiration when building a QMS for AI/ML-CDS usage in the clinic. We finally envision a future in which healthcare institutions run or have access to a medical AI-lab that provides the necessary expertise and quality assurance for AI/ML-CDS implementation and applies a QMS that mimics the ISO15189 used in medical laboratories.

## Introduction

Despite the promise of new digital technologies supporting a more data-driven healthcare system, a significant gap exists between the high number of reported artificial intelligence (AI) and machine learning (ML) based algorithms in academic research and the small number of successfully implemented AI/ML-based clinical decision support (AI/ML-CDS) systems in clinical care. The valorization of AI/ML algorithms into safe and valuable AI/ML-CDS tools is considered a cumbersome process that requires broad in-depth expertise and experience in multiple domains that transcend computer-science and data analysis (1–5).

In 2017, the University Medical Center Utrecht (UMC Utrecht), one of the largest academic teaching hospitals in the Netherlands, started a hospital-wide innovation program to explore if analyses of clinical-care data could be used for AI/ML-CDS-aided personalized care. During this program, several AI/ML-CDS tools were developed in-house and some in co-creation with private parties. In this practice-oriented program, an important lesson learned was the value of a multidisciplinary approach including clinical experts, data scientists, end-users, product/service designers, software engineers, (software) security experts, ethicists, legal experts, financial/business development experts, and change management experts (6). The program evolved into the Digital Health department of the UMC Utrecht, which focuses on accelerating the implementation of digital-health technologies in clinical care for the benefit of our patients.

To support the AI/ML-CDS development process, an innovation funnel geared toward product development for use in clinical care was developed (6) and later served as a blueprint for the development of a national AI innovation tool by the Dutch Ministry of Health (7). The funnel starts with idea generation and ends with implementation in clinical care and transfer of responsibility to operational management. It is divided into seven distinctive phases with transition gates. In each phase, the relevant requirements for the specific phase are addressed including the applicable EU-laws and regulations, existing guidelines, and field standards for AI/ML development, among which are the General Data Protection Regulation (GDPR), Medical Device Regulation/*in-vitro* Diagnostic Regulation (MDR/IVDR), ISO13485 (QMS for the development of medical devices), and IEC62304 (software development lifecycle).

The GDPR, MDR/IVDR, ISO13485 and IEC62304 guidelines and standards are not explicitly developed for AI/ML-CDS tools. Efforts are undertaken to develop standards for AI/ML development (8) and numerous guidance documents exist on how to report AI/ML clinical studies (9–13). Furthermore, in a recent scoping review on guidelines and quality criteria for AI prediction models, it is acknowledged that substantial guidance is available for data preparation, model development, and model validation, while software development, impact assessment, and implementation have received less attention in scientific literature (14). Inspiration for AI/ML-lifecycle management can be gained from approaches such as CRISP-DM/ML (15–17) and contemporary software practices such as DevOps and MLOps (18, 19).

While using the national AI innovation tool as a standardized product development procedure, we have added local AI/ML-description standards, AI/ML-specific standards for version control, AI/ML audits, risk assessments, and ethical assessments. In addition, UMC Utrecht-specific templates and formats have been developed for business case analysis, stakeholder analysis, patient and customer journey analysis, data descriptions, bias risk, and so on. This way, in accordance with the core principles of MDR/IVDR, UMC Utrecht aims to direct the AI/ML-CDS development and implementation process toward a thoroughly controlled standard operating procedure (SOP) to increase the quality of the development process and its delivered products.

The Digital Health department has now progressed to implementing AI/ML-CDS tools in clinical care and this sparked a discussion on how to organize sustainable quality control of AI/ML-CDS tools within the UMC Utrecht, including roles and corresponding responsibilities of the user. ISO13485 and IEC62304 are written from the perspective of the manufacturer and are thus focused on development, implementation, and post-market surveillance procedures of the manufacturer. These guidelines appear less focused on the responsibilities of the user and the implementation of AI/ML-CDS in clinical care. Proper quality assurance requires involvement of both the manufacturer and user.

It struck us that AI/ML-CDS tools, when used as a diagnostic support system, share many similarities with clinical *in vitro* diagnostic tests used in medical laboratories. For *in vitro* devices, input material is urine, blood, or other materials, and the machine is typically a CE-marked chemical analyzer. Likewise, AI/ML-CDS input consists of data and the machine is a software system. Elaborating on this viewpoint, it is our opinion that ISO15189 for medical laboratories may serve as QMS blueprint for operating AI/ML-CDS tools in clinical practice under the MDR or IVDR. This is particularly true when used in conjunction with IEC62304. The interplay between ISO15189 and IEC62304 for software as a medical device (SaMD) under the IVDR has recently been discussed in a paper from our group (20).

In this perspective we illustrate our learnings regarding quality management of AI/ML-CDS tools through an example from our development pipeline, Sleep Well Baby (SWB). After introducing the SWB project and describing the development phase we address life-cycle management questions that arose while operationalizing SWB. When addressing these questions, we illustrate how the organizational structure of medical laboratories and ISO15189 can inspire healthcare institutes in building an effective and sustainable Quality Management System (QMS) for AI/ML usage in clinical care. Finally, in the discussion we provide an outlook how quality management of AI/ML-CDS extends to third-party AI/ML tools and settings outside healthcare institutes other than academic teaching hospitals.

## Sleep-Well-Baby

SWB started as a grassroots project winning the best innovation price at Dutch Hacking Health 2019^1^ It is an in-house developed ML model intended for monitoring real-time sleep-wake patterns in preterm neonates between 28 and 34 weeks gestational age^2^ For the untrained caregiver it is almost impossible to accurately assess the sleep-wake state of preterm infants (21). The added value of real-time sleep-wake state monitoring comes from adapting elective clinical management of these preterm infants toward less disturbance during sleep periods. For a detailed discussion we refer to Sentner et al. (22).

### SWB Development Phase

**Figure 1:**
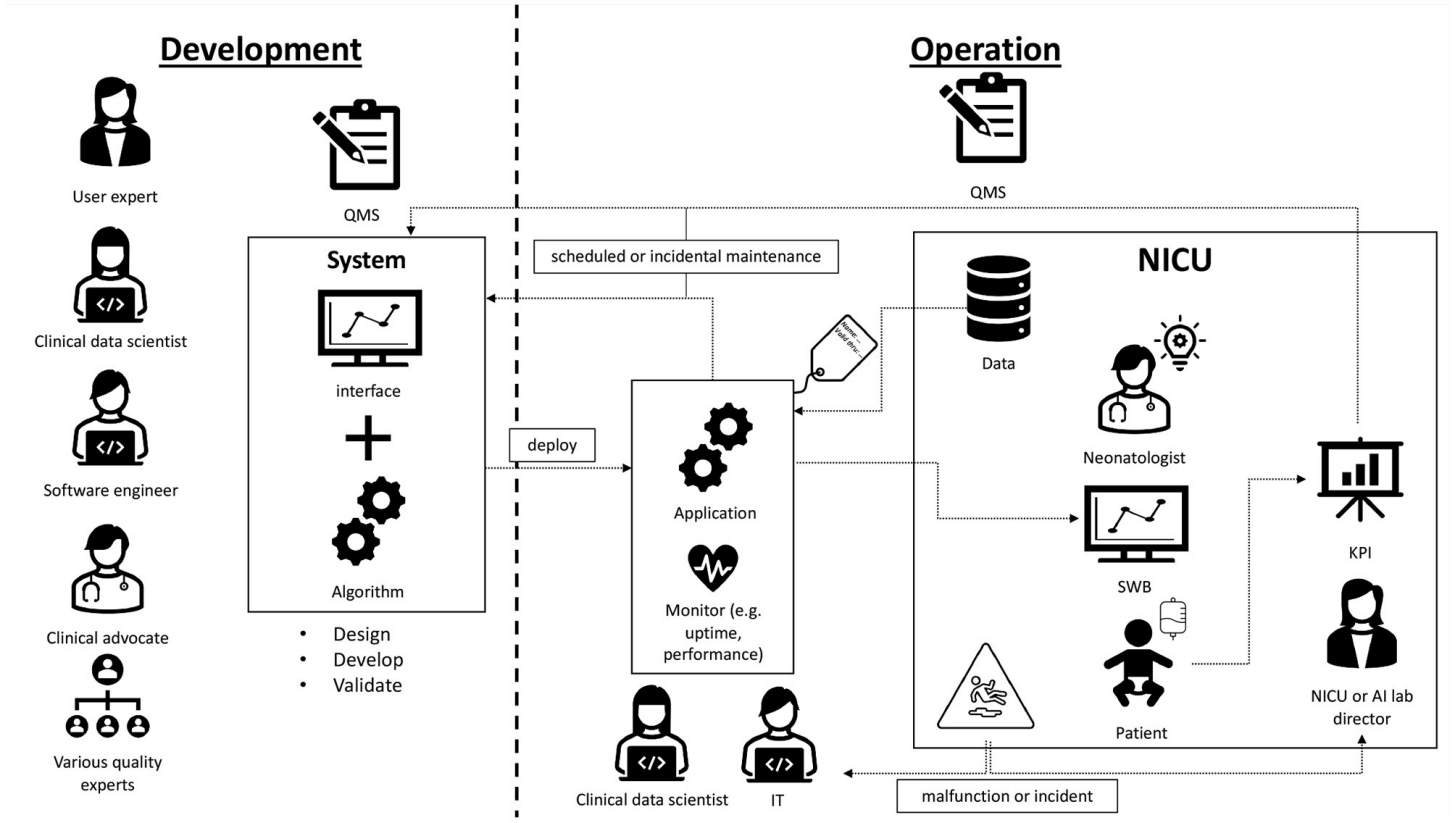
Overview of Sleep Well Baby. Pictorial representation of how SWB was implemented on the NICU of the UMC Utrecht. The algorithm was developed by a multidisciplinary team. Currently, SWB is running bedside. It uses data from the NICU to provide sleep-wake states for preterm infants. The data scientist and software engineer remain involved for troubleshooting, monitoring and continuous maintenance. The director of the NICU is responsible for SOPs regarding AI/ML use. Governance of AI/ML-SaMDs can be done by a central AI lab with a QMS inspired by ISO15189 of the diagnostic laboratory.

SWB was developed following the UMC Utrecht product innovation funnel. According to the MDR it is classified as software as a medical device class 2A, and according to the IEC62304 as category A. Being an in-house developed AI/ML-CDS, it was developed in accordance with art. 5.5 of the MDR where UMC Utrecht is both manufacturer and user. It is running at the NICU of the Wilhelmina Children's hospital (WKZ) in Utrecht and ready for use in clinical impact studies addressing how incorporating sleep-wake state information during clinical care improves patient outcomes. Development was done by a multidisciplinary development team consisting of a clinical expert, several data scientists, ML engineer, user representative and numerous experts in specific fields. During this development process, quality standards including IEC62304, ISO14971, and internal AI/ML standards were applied. Technical and clinical validation was performed by comparing predictions against a ground truth, namely sleep-wake state observations by a highly-trained and internally-calibrated team of students according to a standardized observation method (21). In Figure 1 an overview is given of SWB development and implementation at the NICU of the WKZ. The roles and steps in the development phase are visualized on the left. Moving to the right in the figure the roles and activities in the operational phase are depicted. While transitioning to the operational phase and transferring usage and maintenance responsibilities of the SWB AI/ML-CDS tool to the clinical department, we ran into questions related to SWB life-cycle management that needed answers.

### Who Is Responsible for the AI/ML-CDS Device Configuration?

The SWB configuration was developed involving multiple parties in the UMC Utrecht including the departments of Information Technology (IT), Digital Health and Clinical Physics. Each had a specific role in the development of the device configuration. In summary, the IT department provided the server and platform hosting the model, the Digital Health department data-science team provided the ML application code, and the Clinical Physics department was responsible for real-time extraction of vital parameter data from source instruments. Together with the Digital Health department they arranged the data exchange between source instrumentation, algorithm, and bedside monitor. Finally, they provide the user interface on the bedside monitor for model output. It has been decided that the Digital Health department will serve as the manufacturer and the IT and Clinical Physics departments will serve as subcontractors. The NICU serves as the user. With this division of roles, accompanying responsibilities were established and documented in SOPs and service agreements.

The questions who is responsible for which part of the configuration and who is the manufacturer are crucial in this respect. As variations exist in how AI/ML-CDS tools are configured and hosted, answers may vary per case. For example, a device can be fully developed and hosted by a third-party manufacturer, a UMC Utrecht AI/ML application can be hosted by a third-party, a third-party AI/ML application can be deployed on UMC Utrecht infrastructure, or any other variation. Agreements between parties on for instance maintenance, change management, and support during malfunction need to be addressed using a risk-based approach. ISO15189 contains several norms related to service agreements with suppliers (art. 4.6) and customers (art. 4.4).

### Who Gives Clearance for the Use of SWB in Clinical Practice?

The intended use of SWB was specified by the user, the neonatologist involved. The neonatologist furthermore specified the acceptance criteria and carries responsibility for clearance of the SWB tool. Since clearance requires knowledge about both the healthcare process as well as the AI/ML model performance and its lifecycle, the clinician in charge can bear this responsibility only in consultation with a data scientist who is aware of the medical domain and can assess the device for model performance and lifecycle-management requirements. ISO15189 contains clear guidance on assigning tasks and responsibilities between employees (art 5.1).

The act of formal clearance for use needs to be repeated at specified intervals once the device is in use as part of the regular review cycle and after specific situations in which the performance of the device may be questioned, for example after observed incidents, downtime due to power failure, new releases of supportive software systems, or regular maintenance. Within the UMC Utrecht a record of AI/ML-CDS tools is kept, formal review periods are set, and standard operational qualification procedures are determined using a risk-based approach. ISO15189 contains clear norms for the introduction of equipment (art 5.3.1), reagents and disposables (art 5.3.2) and selection of examination processes (art 5.5.1) which can be extrapolated to introduction of AI/ML-CDS tools in clinical practice.

### How to Ensure Safe Change Management and Revision of SWB?

As part of the development process and before implementation, an extensive risk analysis resembling a health failure mode and effect analysis on the use of the device in the care for patients within the NICU was performed. From this risk analysis agreement was reached between the stakeholders on for example forms of malfunctioning, impact of malfunctioning, and accepted downtimes. ISO15189 contains clear norms on preventive action (art 4.11).

As in-house manufacturer we applied best practices from DevOps to minimize the chance of SWB malfunction and guarantee quick recovery^3^. Change management was done using *git*^4^. Data version control (*dvc*^5^) was used to ensure reproducibility and usage of the correct model in production. SWB code was extensively documented to optimize maintainability and transferability between contributors. Unit and integration tests were written for application code lowering the risk of SWB malfunctioning in clinical practice and ensuring consistency between consecutive releases. Before a change is released it first passes through mandatory review enforced by pull requests. These steps allow semi-automated and fast re-deployment of SWB. When complemented by ISO62304, ISO15189 forms a highly suitable QMS for in-house manufacturing of AI/ML-CDS tools (20).

SWB is an MDR class 2a device and carries limited patient risk. Nevertheless, appropriate procedures and responsibilities must be assigned in the SOPs of the user in case of SWB being temporarily out of service. In our role as manufacturer this implies we have an agreement with the NICU ensuring limited downtime. In practice this means that the software engineer involved in development remains involved to update SWB following the procedures specified above. This specific data science and software engineering knowledge was not transferred to the user. One can imagine that for critical devices (class 3) the user might require 24/7 support and appropriate arrangements within the organization should be established. Again, ISO15189 contains clear norms regarding the management responsibilities in providing resources to ensure quality of provided services (art 4.1.2).

### What if Model Performance Starts Degrading?

Predictive models can degrade over time due to their dependence on input data from potentially changing environments or self-induced feedback loops. Consequently, AI/ML models require monitoring of model performance. During the AI/ML risk analysis, the question was asked: what are the chances of SWB performance degrading? Which process mitigation measures can be applied? And what to do in case of degradation?

SWB is a locked algorithm^6^. Since it only depends on vital parameters, major performance degradation was considered unlikely in the risk analysis. Nevertheless, a change in hardware collecting vital parameters or a changing patient population could result in model drift. The user should be aware of this risk and should be capable to identify it on occurrence. The manufacturer should inform users of this risk in general and specifically in relation to the context in which the AI/ML-CDS tool is used. Building on best-practices from the MLOps movement a monitoring dashboard was designed for SWB, tracking the fraction of valid requests to the model service and tracking distributions of predicted sleep-wake states over time. These distributions serve as a proxy for model performance in absence of a direct accuracy measurement (no other sleep-wake state measurements are performed with regular intervals). Monitoring model performance is, contrary to application performance, not a requirement of the IEC62304, but its relevance is acknowledged (23). In case of degrading model performance, a decision should be made by the user to either (temporarily) terminate the application and/or to re-calibrate and re-validate SWB.

SWB monitoring and re-calibration of the model is done by the Digital Health department since they have the appropriate procedures and competencies. Again, monitoring and re-calibration requires the expertise of data scientists. Furthermore, since UMC Utrecht is the manufacturer and user, we have access to the required data to perform monitoring. However, for most manufacturers this will not necessarily be the case. In this situation the manufacturer could make available tooling for monitoring and re-calibration, or the user should set up monitoring procedures themselves. Figure 1 on the right depicts the continuous involvement of the data scientist in monitoring the application.

### Who Provides a Helpdesk for Users?

Sections How to ensure safe change management and revision of SWB? and What if model performance starts degrading? discussed malfunction and model degradation. This raises the question, what if a user experiences a malfunction? Or what if an incident involving SWB occurs? The user is responsible for having appropriate incident management, in addition to the post market surveillance responsibilities of the manufacturer. Feedback of incidents affecting patient care is already covered by existing NICU procedures. For malfunctions not directly affecting the patient a SWB helpdesk was created. Here reports can be filed and will be handled by the appropriate experts, such as described in the previous section and illustrated in Figure 1.

### How Are Users Trained?

A prospective risk analysis performed by the user revealed the risk of SWB being incorrectly used due to imperfect model performance and raised the question: how can this be prevented? SWB is a sleep-wake monitoring system intended primarily for nurses to plan elective care (e.g., changing diapers). It differs from other monitors-such as heart rate-in that it is not based on direct physiological measurement but instead makes a prediction with imperfect precision. In addition, it was developed for a particular population of preterm infants, i.e., inclusion criteria. Nurses and neonatologists should be aware of these limitations such that they can use the device appropriately. The NICU should ensure appropriate SOPs for SWB, including procedures on disregarding SWB advice. Meanwhile, the manufacturer should provide user instructions and guidance documentation specifying amongst other things the intended use, mode of operation, intended patient population and limitations in terms of sensitivity and specificity. This is similar to instructions included with medication or *in vitro* devices. User-employed specialists or the manufacturer should provide training and guidance to end-users when required. In the medical lab it is customary to organize a training by the manufacturer with the introduction of a new analyzer. After the introduction of the analyzer new employees are trained internally by internal employees who are competent in operating the analyzer. ISO15189 provides clear norms on training programs for employees (5.1.5) and monitoring and assessing competences of employees (5.1.6) which can be extrapolated to AI/ML-CDS usage.

## Discussion and Conclusion

In the context of SWB, we discussed a selection of quality aspects and responsibilities that surface when operating AI/ML-CDS in clinical practice. We showed how ISO15189 can be a source of inspiration for a healthcare institute its QMS for operating and in-house manufacturing of AI/ML-CDS tools. UMC Utrecht is learning-by-doing, SWB is only a first example and the effort of implementing quality measures to ensure safe use of AI/ML-CDS tools in clinical practice is still in progress. Moreover, the AI/ML field itself is still maturing and quickly evolving.

SWB is an in-house developed ML algorithm where UMC Utrecht is both manufacturer and user. The extrapolation to AI/ML purchased from a third-party is relatively straightforward. Manufacturers should adhere to a QMS for production such as ISO13485. Users of third-party devices are accountable for responsible use of AI/ML-CDS, their QMS should include processes for selection, clearance and performance verification, appropriate SOPs, and service agreements with the manufacturer relating to monitoring and change management. ISO15189 could provide inspiration for this. It is of great importance that the user has the appropriate expertise to audit (24) and validate AI/ML-CDS tools or else a situation can arise where underperforming and potentially harmful use of AI/ML in clinical practice is not being identified (25). In case departments of a healthcare institution are unable to provide this expertise themselves, it could be bundled in a centralized AI laboratory.

Our recommendations hold true for larger healthcare institutions such as academic teaching hospitals who can build the necessary resources and competences needed for safe operation of AI/ML-CDS tools. For smaller entities, such as a single general practitioner, this effort seems unfeasible. In this situation, complete dependence on the manufacturer is imaginable, making it difficult to establish truly safe performance. Again, inspiration can be found in the regional services of medical laboratories that very often provide access to competences and resources for safe application of diagnostics. Regional AI labs could provide services for the development, acquisition, and quality control of AI/ML for smaller healthcare institutes including general practitioners. Like medical laboratories they could educate and assist healthcare professionals in the selection and safe use of AI/ML.

Complying with an extensive user QMS is time-intensive, expensive, and might appear to hamper innovation. However, just like *in vitro* devices, an appropriate QMS is a necessity for safe AI/ML use within healthcare settings. In spirit with the MDR/IVDR it is quality first. Moreover, so far AI/ML has not yet lived up to its promise to revolutionize healthcare. Although we believe it has the potential to do so, we do not envision a disruptive change in which dozens of AI/ML-CDS systems will independently enter every department in the coming years. Instead, it will more likely be a regulated introduction similar in pace to the way new *in vitro* devices or medication are introduced. We strongly believe an appropriate QMS will not only guarantee safe use, but also helps accelerate implementation. The lessons learned and identified quality criteria in this perspective illustrate that ISO15189 can serve as an inspiration and provide a starting point for organizations building their own data-driven capacity to improve patient care.

## Data Availability Statement

The original contributions presented in the study are included in the article/supplementary material, further inquiries can be directed to the corresponding author.

## Author Contributions

RB, DO, and AV initiated this perspective. JD is the neonatologist in charge of the SWB project. RB and AV drafted the manuscript SH provided extensive feedback on the manuscript. All authors took part in discussion, revision of the manuscript, contributed to the article, and approved the submitted version.

## Conflict of Interest

The authors declare that the research was conducted in the absence of any commercial or financial relationships that could be construed as a potential conflict of interest.

## Publisher's Note

All claims expressed in this article are solely those of the authors and do not necessarily represent those of their affiliated organizations, or those of the publisher, the editors and the reviewers. Any product that may be evaluated in this article, or claim that may be made by its manufacturer, is not guaranteed or endorsed by the publisher.

## Footnotes

^1^ https://dutchhackinghealth.nl/

^2^At the time of writing the bedside implementation of SWB is still in the process of being clinically verified.

^3^The DevOps movement is the current paradigm in software development, combining development (Dev) and operations (Ops) teams for increased efficiency throughout the software lifecycle (18). IEC62304 is sometimes believed to hamper the use of contemporary software development practices such as DevOps (26). However, we believe that agile DevOps change management practices can be successfully combined with the MDR and IVDR, which prescribe the use of generally acknowledged state-of-the-art technologies. Moreover, we are of the opinion that activities prescribed by IEC62304 and the quality control measures they enforce can be successfully incorporated within the DevOps philosophy.

^4^ https://git-scm.com

^5^ https://dvc.org

^6^There is a distinction between locked and adaptive algorithms. Locked algorithms are static functions. SWB is a static classifier, given the same input data it will always return the same result. A locked algorithm can be re-calibrated or updated manually in an *ad-hoc* fashion, for example when introduced to a new ward or when a larger dataset becomes available. On the contrary, adaptive algorithms are continuously updated through a (semi-)automatic process. In theory, such algorithms can adapt automatically to a changing environment to prevent model drift.

## Abbreviations

AI: artificial intelligence
CDS: clinical decision support
CRISP-DM: cross industry standard process for data mining
GDPR: general data protection regulation
IVDR: *in-vitro* diagnostic regulation
MDR: medical device regulation
ML: machine learning
NICU: neonatal intensive care unit
QMS: quality management system
SaMD: software as a medical device
SOP: standard operating procedure
SWB: sleep well baby.

## Nomenclature

### International Organization for Standardization (ISO) Norms

IEC 62304:2006+A1:2015

Medical device software–Software life cycle processes

ISO 13485:2016

Medical devices–Quality management systems–requirements for regulatory purposes

ISO 15189:2012

Medical laboratories–Requirements for quality and competence.
